# Using the integrative model of behavioural prediction to understand COVID-19 vaccine hesitancy behaviour

**DOI:** 10.1038/s41598-022-12466-0

**Published:** 2022-06-04

**Authors:** John Romate, Eslavath Rajkumar, Rajgopal Greeshma

**Affiliations:** Department of Psychology, Central University of Karnataka, Kalaburagi, Karnataka India

**Keywords:** Psychology, Human behaviour

## Abstract

The officials realized that the vaccination drive alone would not be  sufficient, but the individual's response towards getting vaccinated needs to be assessed and addressed, especially in India, where the diverse culture could widely affect the population's vaccination behaviour. The study aimed to identify the predictors of vaccine hesitancy behaviour using the health belief model and theory of planned behaviour and understand mediating and moderating influence of knowledge and social support on the relationship between the predictors and vaccine hesitancy behaviours among the Indian population. Data was collected from 1006 samples. Regression analysis was performed to assess the variances exerted on vaccine hesitancy behaviours. Also, SEM AMOS was employed to examine the mediation and moderation effects of knowledge about vaccines and social support. The findings indicated that around 11% of the respondents were hesitant to get vaccinated. The combined models of HBM and TPB provide high predictive power. The analysis also revealed that knowledge about vaccine significantly mediates partially between a few constructs of HBM and TPB concerning hesitancy. This study provides the theoretical framework and suggests that the health belief model and the theory of planned behaviour model could explain the psychological influences of vaccine hesitancy in India.

## Introduction

Globally, as of 9th July 2021, 185,291,530 coronavirus cases and nearly 4,010,834 deaths were reported to the World Health Organization^1^. In India, the caseloads have constantly been rising, with 30,752,950 total cases and 405,939 deaths until the 9th of July 2021^1^. Currently, Covishield and Covaxin are widely administered vaccines in India^2^. Covishield and Covaxin were first administered on 16th January 2021^1^. Although, India started the vaccination drive well by being able to provide one of the fastest and largest vaccination campaigns in the world^3^, it eventually came down to an average speed, with the government facing criticisms over the management of the second wave of COVID-19 in India and the delay in rolling out of the vaccines^4^.

Although the government has initiated its vaccination drive programme, the officials are constantly challenged to convince the public to get vaccinated, one of the reasons being vaccine hesitancy^5^ which refers to the individuals’ delayed response or refusal to get vaccinated despite the availability of vaccines^6^, hence resulting in failure of mass immunization, despite the government’s efforts to providing vaccines to communities. Thus, to understand the mechanisms of such hesitancy behaviour, a model in the literature with its centrality and relevance sounds greatly emphasize elaborating the behaviours and guides the efficient ways to tackle the issues of hesitancy, especially about vaccines in this context. A ‘3Cs’ model was put forth^7^ explaining the complexity of hesitancy. The 3Cs represented three different categories viz., complacency, convenience and confidence^6^. Complacency is likely when the perceived risks concerning disease-preventable vaccines are low, and thus the need for vaccine becomes not mandatory. Consequently, the potential risks involved in vaccination of specific illnesses are self-decided by individuals, resulting in their hesitancy towards the same, especially when the vaccines are perceived to be less effective in tackling the upcoming consequences. Convenience deals with the physical constructs that could affect an individual’s decision to be immunized. Thus, the availability of vaccines, geographical conditions, affordability, literacy, and quality of health services would influence an individual’s vaccine hesitancy behaviour. The third category, confidence, represents the trust that the individuals put forth in the efficacy and need for vaccinations, the health workers and services involved in the immunization process, and the policy workers involved in the decision making process concerning vaccine uptakes^6^.

While the 3C model incorporates the perspectives required to understand vaccine hesitancy, multiple factors could be influencing vaccine hesitancy. Various behaviour change models tried to explain why people engage in a particular behaviour. Among them, the health belief model (HBM) is a widely used model that aids in investigating the otherwise ignored factors, thus bridging the gap between health behaviour and the use of health services^8^. Health behaviours are activities people engage in to prevent themselves from infections^9^. The individuals’ perception of susceptibility to infection, their perception of the severity of the virus, the kind of barriers they perceive, and the benefits they perceive are involved in behaviour change. Besides, various internal and external motivating cues encourage behaviour changes and self-efficacy^10^. This health-specific social cognition model, therefore, explains the individual’s belief concerning the COVID-19 disease^9,11,12^, which would also aid in predicting their vaccine hesitancy behaviour.

Specifically, perceived susceptibility from the HBM model indicates the individual’s perception of the possibility or the extent of contracting a disease or virus. Consequently, the dimension should explain the hesitancy behaviour, or highlight the individual’s perception of the virus. Likewise, perceived severity emphasizes the physical, mental and or social problems that the disease would induce on the individual if infected. While perceived barriers highlight the issues or difficulties that the individuals would confront when agreeing to get vaccinated, perceived benefits indicate the positive consequences they would have gained or experienced by deciding to get vaccinated^13^. Although HBM tries to incorporate various factors that influence health-related behaviours, in this case of vaccine hesitancy, it does not account for an individual’s attitude or intention in executing the health behaviours^8,9,11,12^. Moreover, literature does justify that it would be unfair to not look into other factors that would plausibly influence hesitancy^10^. By recognizing that other theories could influence equally and thus could be incorporated in behaviour change intervention, the theory of planned behaviour (TPB) is also being utilized in the current study to explore its influence among the Indian population.

The TPB model also is a widely used theoretical construct that aids in answering various health-related research gaps from a social cognitive perspective. The significant or core aspects of TPB are intention and behaviour. The intention has been further represented and explained with three main dimensions: attitudes, subjective norms, and perceived behavioural control. While attitude explains the assessment of negative and positive outcomes of the behaviour, subjective norm explains the perception of the support extended from the family and known individuals that would positively influence the behaviour change. Perceived behaviour control describes how one perceives their capability to undergo or act on implementing the behaviour change^14^. In addition, a new component, anticipated regret, has also been added and studied in the current study due to its predictive property^15,16^. Researchers have also emphasized the negative emotional arousal that regret brings forth when individuals fail to act in a certain way and begin to think counterfactually. Thus, anticipating regret may probably influence individuals to adopt a specific behavioural change^15^.

Notably, studies have integrated HBM and TPB models to explain specific health-related behaviours, including vaccine hesitancy^8,12,17^. As opposed to other models such as the transtheoretical model (TTM), social norms theory, social cognitive theory (SCT), etc.^11,18^, the integration of HBM and TPB models would provide a more holistic perspective concerning the beliefs and intentions the individuals hold on, especially concerning vaccine hesitancy^8,12,19^. Furthermore, other models seem inappropriate in comprehensively attending to factors predicting vaccine hesitancy, including models like TTM, which are better applicable towards designing public health interventions that emphasise and monitor the individual’s decision-making process at every stage^18,19^. SCT model, on the other hand, emphasizes on maintaining behaviours and helping individuals to regulate their goal-directed behaviours through reinforcements and control^18^. However, the present study context aims to emphasise the factors that would initiate the outcome behaviour of vaccine hesitancy. Therefore, HBM and TPB frameworks together seem to provide a better model for explaining health behaviour changes concerning vaccine uptake^8,12,19^. Previous literature also emphasized the role of HBM and TPB models in predicting behavioural changes during the MERS-Cov outbreaks^20–22^. However, these studies are more prominent in Western countries but not in India. Moreover, the impact of the second wave of COVID-19 in India has encouraged researchers to identify the possible behavioural causes for the virus to escalate. More importantly, there is a lack of adequate understanding and consensus regarding the influence of psychological factors on vaccine hesitancy in India, resulting in a reduced number of fully vaccinated individuals compared to the US and UK populations^23^.

To add on, some general surveys have indicated that in India, only around 5% of the people have been fully vaccinated due to vaccine hesitancy which is more or less influenced by misconceptions and misinformation regarding vaccines and their side effects, particularly the Indian cultural and religious beliefs^24^. Therefore, using both HBM and TPB would contribute to the current knowledge concerning the Indian sample characteristics. Although the government has initiated vaccination drives and campaigns, the percentage of vaccine intake has been reduced. The acceptance rate is low in both urban and rural Indian populations^23,24^, leaving the government and health authorities equivocal and perplexed. To tackle these issues, the Government introduced plans to reach out to influential political leaders, actors, *Sarpanch* (village level heads) and religious leaders and thereby to educate them about the safety, effectiveness and necessity of availing COVID-19 vaccination as per the WHO guidelines. These influential groups could then create awareness among the people at different district and state levels in the country. This social mobilization plan eventually yielded positive results with fewer vaccine hesitant people^25,26^. However, though these social campaigns are developing, it is essential to explore the predictors of vaccine hesitancy through the current study, which could be incorporated in the social mobilization plans and also used by the role model to influence the general public in vaccine uptake behaviours.

India being varied and diverse in socio-economic and literacy factors, this study would represent a unique approach as it will integrate the HBM and TPB models and explore the role of knowledge and social support. The main intention of this correlational research study is to gain deep level consensus from the general Indian population concerning the predictors of vaccine hesitancy in India and the role of knowledge and social support in vaccine acceptance. The study would help build a theoretical or conceptual framework around the health belief model and the theory of the planned behaviour, which have proved to contribute as strong predictors in other research studies that exclude India^16,27^.

## Methods

### Design

The present research utilized a correlational research design to explore the study objectives. The main objectives were (1) to determine the prevalence of COVID-19 vaccine hesitancy behaviour, (2) to identify the predictors of vaccine hesitancy behaviour using the health belief model and the theory of planned behaviour, and 3) whether knowledge and social support mediate or moderate the relationship between the predictors and vaccine hesitancy. The current study received ethical approval from the School of Social and Behavioral Sciences, Central University of Karnataka, India (Ethical clearance number: CUK/SDBD/Psy/EC-15/2020-21/15, Dated 09-06-2021). Additionally, the study methods followed all the relevant guidelines and regulations.

### Participants

The study utilized convenient sampling to recruit the 1006 participants via an online portal. The data collection was initiated in July, which proceeded for approximately two months. The participants above 18 years of age and willing to participate in the study were included, while individuals unwilling to participate were excluded from the study. The study also excluded individuals with mental illness which was confirmed in the online survey through self-report questions such as “*whether the individual was seeking any psychological help in the previous recent months? Was the individual on any medications concerning mental health issues in the recent months?*” Informed consent was obtained from the participants before the initiation of the study. Most of the participants were from states of India such as Karnataka (31.9%), Tamil Nadu (15.50%), Kerala (22.76%), Telangana (7.15%), Andhra Pradesh (8.7%), Maharashtra (1.98%), Delhi (6.66%), Uttar Pradesh (1.88%), Rajasthan (1.29%), Haryana (1.09%) and Punjab (0.99%). Also, the data collected included vaccinated and unvaccinated participants (Table 1). The data regarding vaccine uptake was gathered to understand the percentage of individuals who have been vaccinated with a minimum of one dose^28^. Nearly 1.19% were fully vaccinated, while 9.84% were partially vaccinated.

**Table 1:** Distribution of socio-demographic characteristics of the participants.

Variables	n (1006)	n%
**Gender**		
Male	371	37
Female	632	63
**Religion**		
Hindu	520	51.7
Muslim	64	6.4
Christian	353	35.1
Prefer not to say	54	5.4
Others	15	1.5
**Education**		
Higher secondary	50	5
High school	50	5
Diploma	30	3
Under Graduation	415	41.3
Post-Graduation	390	38.8
MPhil and PhD	71	7.1
**Marital status**		
Unmarried	818	81.3
Married	185	18.4
Divorced	3	0.3
**Occupation**		
Student	576	57.3
Unemployed	162	16.1
Employed	268	26.6
**Socio Economic Status**		
Low	70	7
Middle	900	89.5
High	36	3.6
**Residence**		
Urban	316	31.4
Rural	390	38.8
Semi Urban	299	29.8
**Region**		
Karnataka	321	31.9
Tamil Nadu	156	15.50
Kerala	229	22.76
Andhra Pradesh	88	8.7
Telangana	72	7.15
Maharashtra	20	1.98
Delhi	67	6.66
Uttar Pradesh	19	1.88
Rajasthan	13	1.29
Haryana	11	1.09
Punjab	10	0.99
**Vaccine details**		
Fully Vaccinated	12	1.19
Partially Vaccinated	99	9.84
Unvaccinated	895	88.96

### Survey instruments and statistical techniques

Along with the socio-demographic information of the participants, detail concerning their vaccine intake was also obtained through the survey. The current study used two items adapted from a study by Hossain et al.^16^ to assess vaccine hesitancy: (a) If you get the chance of getting a COVID-19 vaccine for free, what will you do? (b) If your family or friends think of getting the COVID-19 vaccine, what will you do?. These items used a 6-point Likert scale (1 = surely will take it. 6 = surely I will not) with an internal consistency of Cronbach’s alpha − 0.843. In addition, HBM’s six dimensions^12^ viz. perceived susceptibility, perceived severity, perceived benefits, perceived barriers, cues to action, and self-efficacy were assessed using a 5-point Likert scale (1 = strongly disagree to 5 = strongly agree). Similarly, the TPB constructs^16^ viz. attitude toward a vaccine, subjective norm, perceived behavioural control, and anticipated regret were assessed using a 5-point Likert scale (1 = strongly disagree to 5 = strongly agree). Additionally, for the present study, knowledge about the vaccine (1 = strongly disagree to 5 = strongly agree), and social support (1 = strongly disagree to 5 = strongly agree) were considered as the mediating and moderating variables with internal consistencies of (Cronbach’s alpha) 0.626 and 0.70 respectively.

To analyze the results based on the study's objectives, correlation and multiple linear regression analyses were conducted using IBM SPSS 25 software. Multiple linear regression was used to understand the predictive power of the integrated models of HBM and TPB. Moreover, mediation and moderation analysis were conducted using AMOS software v25.0 to examine the mediating effects of knowledge about vaccine and moderating effects of social support. The assumptions of normality, linearity, homogeneity and multicollinearity for the statistical tests were met. A test of normality was performed using the p–p plot^29^ and was found to be normally distributed. The linearity between the variables was achieved by inspecting the scatterplots^30^, Homogeneity of variance or homoscedasticity was tested by creating the scatterplots between the residuals with the dependent variable. Multicollinearity was tested using the VIF values and was found to be below 10, thereby meeting the assumption. Finally, the assumption for auto-correlation was also met with the Durbin–Watson test^29,30^.

## Results

Analysis revealed the socio-demographic characteristics of the participants (Table 1). About 63% of the sample consisted of females, 37% were males. Likewise, most individuals have basic educational qualifications.

The findings (Table 2) indicated that around 89% were willing to be vaccinated (i.e.,75.8% surely will take it and 13.2% probably will take it) while 3.9% delayed taking it. 4% were undecided, and 3.1% would reject taking the vaccine even if provided for free (i.e., 1.6% probably will not take it and 1.5% surely will not take it). Vaccine hesitancy refers not only to the refusal of the vaccine but also delay in the acceptance of vaccines despite the availability of free vaccines^6^. Thus combining the results of delayed and undecided participants would not affect the interpretation of the results. Therefore, when we consider the unsure participants and those who delay the vaccine intake, the findings revealed that around 11% of the respondents were hesitant to vaccinate. Similarly, vaccine-hesitant behaviours were also observed among respondents’ support towards their family and friends in getting vaccinated (Table 3). 92% would encourage their family and friends to get vaccinated (i.e.,70.8% strongly encourage and 21.2% encourage them), while 8.1% showed vaccine hesitancy behaviours concerning their family and friends’ decision to get vaccinated (i.e., 2.4% convince to delay, 4.4% no action and 1.3% strongly discourage them).

**Table 2:** Frequency distribution of participants’ opinion concerning their vaccine intake.

Opinion about intake of vaccine	n (1006)	n%
Surely will take it	763	75.8
Probably will take it	133	13.2
Will delay taking it	39	3.9
Not sure	40	4.0
Probably will not take it	16	1.6
Surely will not take it	15	1.5

**Table 3:** Frequency distribution of the opinion of the participants about the willingness of their friends or family to be vaccinated.

Participants’ opinion about their family members or friends to get vaccinated	n (1006)	n%
Strongly encourage them	712	70.8
Encourage them	213	21.2
Ask them to delay getting the vaccine	24	2.4
Will not say anything about it	44	4.4
Strongly discourage or forbid them from taking vaccine	13	1.3

Table 4 represents the correlational analysis of the study variables with vaccine hesitancy. Except for perceived susceptibility and perceived severity, all other constructs of HBM have significant relationships with vaccine hesitancy. Perceived benefits, cues to action, and self-efficacy negatively correlated with vaccine hesitancy, while perceived barriers positively correlated with hesitation. Perceived benefits were found to be indicating the highest relation with vaccine hesitancy followed by self-efficacy. Contrarily, all the constructs of TPB have significant associations with vaccine hesitancy. A negative attitude towards vaccine has a positive  relation with vaccine hesitancy, while subjective norms, perceived behavioural control, and anticipated regret are negatively correlated with vaccine hesitancy. Of these, negative attitude toward vaccine was found to be having the highest correlation with vaccine hesitancy. Additionally, knowledge about vaccine was also found to have a significant negative relationship with vaccine hesitancy.

**Table 4:** Correlation matrix: domains of health belief model and domains of the theory of planned behaviour and vaccine hesitancy.

	Vaccine hesitancy	Mean	SD
Social support	− 0.191**	8.33	1.62
Perceived susceptibility	− 0.047	5.76	2.05
Perceived severity	0.003	5.95	2.18
Perceived benefits	− 0.424**	12.22	2.60
Perceived barriers	0.122**	12.62	3.61
Cues to action	− 0.318**	16.06	3.07
Self-efficacy	− 0.383**	11.91	2.53
Knowledge about vaccine	− 0.295**	13.86	2.97
Negative attitudes towards vaccine	0.425**	11.62	5.42
Subjective norms	− 0.300**	8.56	1.99
Perceived behavioural control	− 0.328**	4.33	0.940
Anticipated regret	− 0.188**	3.60	1.312

**p < 0.01.

The multiple regression analysis (Table 5) infers that 29.8% (Adjusted R square = 0.291) of variance in COVID-19 vaccine hesitancy is exerted by the combined dimensions of HBM and TPB. Of the HBM dimensions, perceived susceptibility (β = − 0.066, SE = 0.029, *p* < 0.05), perceived benefits (β = − 0.159, SE = 0.025, *p* < 0.001), perceived barriers (β = 0.071, SE = 0.015, *p* < 0.05) and self-efficacy (β = − 0.110, SE  = 0.025, *p* < 0.01) were the significant predictors of vaccine hesitancy. Additionally, of the TPB dimensions, negative attitude towards vaccine (β = 0.254, SE = 0.011, *p* < 0.001) and anticipated regret (β = − 0.060, SE = 0.039, *p* < 0.01) were significant predictors of vaccine hesitancy. These findings also indicate that an increase in perceived susceptibility, perceived benefits, self-efficacy and anticipated regret leads to a decrease in vaccine hesitancy. In contrast, an increase in perceived barriers and negative attitude towards vaccine leads to increased vaccine hesitancy. Additionally, a negative attitude towards vaccine was found to be exerting the highest variance on vaccine hesitancy followed by perceived benefits. Therefore, a unit increase in a negative attitude towards vaccines would increase the vaccine hesitancy by 0.254 units, whereas a unit increase in perceived benefits would lead to a 0.159 units reduction in vaccine hesitancy.

**Table 5:** Multiple linear regression analysis—the six domains of health belief model and four domains of the theory of planned behaviour as a predictor of vaccine hesitancy.

Variables	Model 1 Std β	SE	t
Perceived susceptibility	− 0.066	0.029	− 1.972*
Perceived severity	0.015	0.028	0.436
Perceived benefits	− 0.159	0.025	− 4.439***
Perceived barriers	0.071	0.015	2.344*
Cues to action	− 0.054	0.019	− 1.615
Self-efficacy	− 0.110	0.025	− 3.101**
Negative attitude towards vaccine	0.254	0.011	7.490***
Subjective norm	− 0.047	0.030	− 1.395
Perceived behavioural control	− 0.060	0.067	− 1.719
Anticipated regret	− 0.060	0.039	− 2.142**
R	0.546		
R^2^	0.298		
F	42.227***		
ΔR^2^	0.291		
ΔF	42.227		

HBM dimensions: perceived susceptibility, perceived severity, perceived benefits, Perceived barriers, cues to action and self-efficacy; TPB dimensions: Negative attitude towards vaccine, subjective norms, perceived behavioural control and anticipated regret; Std β—standardised beta or regression co-efficient; t—t value, R—represents the correlation; R^2^—variance in the outcome explained in the model; F—F ratio.

*p < 0.05; **p < 0.01; *** p < 0.001.

**Figure 1:**
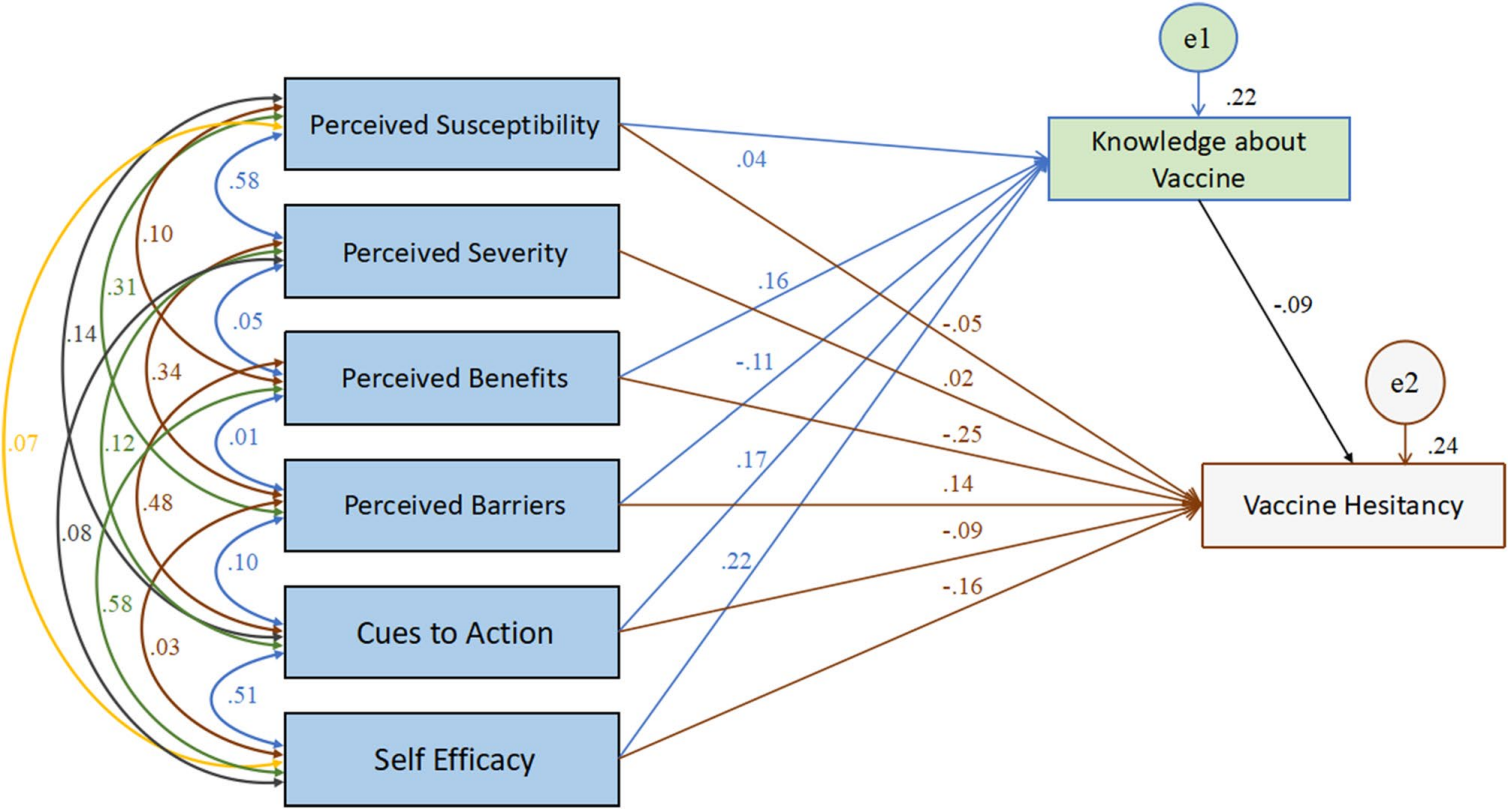
The mediating effects of knowledge about vaccines on the relationship between HBM constructs and vaccine hesitancy.

AMOS software v25.0 was utilized to investigate the mediation and moderation effects of knowledge about vaccine and social support on the relationship between predictor and criterion variables. According to the goodness-of-fit statistics, both the HBM and TPB models showed good fit (For the HBM model, CMIN/DF = 0.147 with probability value = 0.701 which is higher than 0.05; RMSEA = 0.000; GFI = 1.000; AGFI = 0.999; RFI = 0.998; CFI = 1.000; For TPB model, CMIN/DF = 3.387 with probability value = 0.066; RMSEA = 0.049; RFI = 0.955; CFI = 0.998). For the HBM model, to obtain a good-fit, the model was adjusted by removing the path from perceived severity (which were not significant) to the mediating variable viz, knowledge about vaccine as suggested by the AMOS output. Although, the path from perceived susceptibility also were not significant, when run an alternative model without the dimensions of perceived severity and perceived susceptibility, most of the fit indices were 1, giving a perfect fit. Consequently, AMOS output showed that the probability level could not be computed as there were zero degrees of freedom (df = 0). Although the model provides meaning, but no prediction model would be this good^31^. The information was exactly equal to the number of paths needed to be estimated which lead for the model to be “just-identified”^31^. Therefore, the dimensions were retained and the suggested path by the AMOS output viz, the path from perceived severity to knowledge about vaccine only was removed, leaving us with a good fit model. Further, the analysis (Table 6, Fig. 1) obtained from the AMOS output revealed that the knowledge about vaccine significantly mediates partially (direct effects are significant as well) on the relationship between perceived benefits and vaccine hesitancy (β = − 0.011, Std β = − 0.015, SE = 0.006, *p* < 0.01), perceived barriers and vaccine hesitancy (β = 0.005, Std β = 0.010, SE = 0.005, *p* < 0.01), cues to action and vaccine hesitancy (β = − 0.010, Std β = − 0.016, SE = 0.006, *p* < 0.01), and self-efficacy and vaccine hesitancy (β = − 0.014, Std β = − 0.020, SE = 0.008, *p* < 0.01) but did not mediate between perceived severity and hesitancy, and perceived susceptibility and hesitancy. These values indicated that the largest indirect effect for vaccine hesitancy were by self-efficacy with β = − 0.014, Std β = − 0.020, *p* < 0.01. In addition, perceived benefits had the highest direct effect on hesitancy (β = − 0.174, Std β = − 0.252, SE = 0.036, *t* = − 7.0757, *p* < 0.05), followed by self-efficacy (β = − 0.110, Std β = − 0.154, SE = 0.037, *t* = − 4.2699, *p* < 0.01). Furthermore, perceived benefits had the highest total effects on vaccine hesitancy (β = − 0.185, Std β = − 0.268, SE = 0.035, *t* = − 7.5563, *p* < 0.05), followed by self-efficacy (β = − 0.124, Std β = − 0.174, SE = 0.036, *t* = − 4.8908, *p* < 0.01).

**Table 6:** The mediating effects of knowledge about vaccine on the relationship between HBM constructs and vaccine hesitancy.

Path	β	Stdβ	SE	95% CI [LCI, UCI]	*t*	*P*
**(a) Mediation effect of knowledge about vaccine on the relationship between perceived benefits and vaccine hesitancy**						
Total effect	− 0.185	− 0.268	0.035	− 0.337, − 0.194	− 7.5563	0.013
Direct effect	− 0.174	− 0.252	0.036	− 0.320, − 0.180	− 7.0757	0.020
Indirect effect	− 0.011	− 0.015	0.006	− 0.029, − 0.005	–	0.006
**(b) Mediation effect of knowledge about vaccine on the relationship between perceived barriers and vaccine hesitancy**						
Total effect	0.076	0.153	0.030	0.094, 0.209	5.0174	0.013
Direct effect	0.071	0.143	0.030	0.087, 0.201	4.6749	0.016
Indirect effect	0.005	0.010	0.005	0.004, 0.022	–	0.008
**(c) Mediation effect of knowledge about vaccine on the relationship between cues to action and vaccine hesitancy**						
Total effect	− 0.065	− 0.110	0.033	− 0.190, 0.236	− 3.2986	0.014
Direct effect	− 0.055	− 0.094	0.033	− 0.172, − 0.032	− 2.7893	0.014
Indirect effect	− 0.010	− 0.016	0.006	− 0.030, − 0.006	–	0.005
**(d) Mediation effect of knowledge about vaccine on the relationship between self-efficacy and vaccine hesitancy**						
Total effect	− 0.124	− 0.174	0.036	− 0.239, − 0.093	− 4.8908	0.009
Direct effect	− 0.110	− 0.154	0.037	− 0.224, − 0.076	− 4.2699	0.012
Indirect effect	− 0.014	− 0.020	0.008	− 0.037, − 0.006	–	0.004

All parameters obtained from the AMOS output with the HBM dimensions: perceived susceptibility (not significant), perceived severity (not significant), perceived benefits, Perceived barriers, cues to action and self-efficacy; β—unstandardised beta co-efficient; Stdβ—standardised beta co-efficient; SE—standard error; 95% CI—95% confidence interval with lower bounds and upper bounds [LCI, UCI]; t—t value, *p*—shows the significant level.

**Figure 2:**
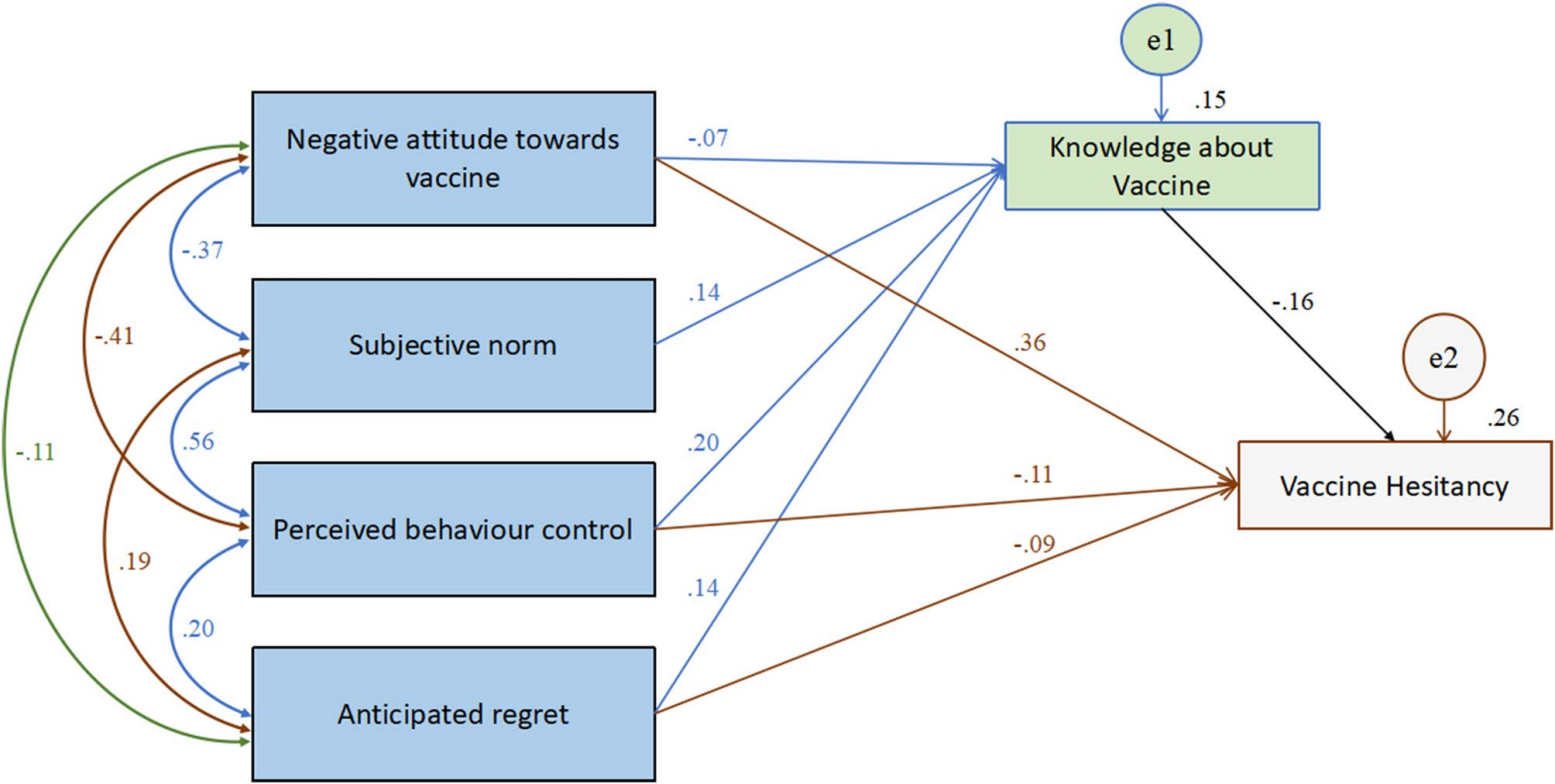
The mediating effects of knowledge about vaccines on the relationship between TPB constructs and vaccine hesitancy.

Besides, in the TPB model as well (Table 7, Fig. 2), knowledge about vaccine significantly mediates partially (direct effects are significant as well, except for subjective norm) the relationship between all the constructs of TPB and vaccine hesitancy: negative attitude toward vaccine and vaccine hesitancy (β = 0.004, Std β = 0.011, SE = 0.008, *p* < 0.05), subjective norm and vaccine hesitancy (β = − 0.020, Std β = − 0.022, SE = 0.009, *p* < 0.01), perceived behavioural control and vaccine hesitancy (β = − 0.060, Std β = − 0.031, SE = 0.009, *p* < 0.01), and anticipated regret and vaccine hesitancy (β = − 0.031, Std β = − 0.022, SE = 0.007, *p* < 0.05). Of these indirect effects, perceived behaviour control had the highest indirect effect on vaccine hesitancy (β = − 0.060, Std β = − 0.031, *p* < 0.01). Additionally, it was found that perceived behaviour control had the highest direct effect on vaccine hesitancy (β = − 0.204, Std β = − 0.106, SE = 0.043, *t* = − 2.2515, *p* < 0.01) followed by anticipated regret with β = − 0.127, Std β = − 0.092, SE = 0.026, *t* = − 3.1454, *p* < 0.01. However, the direct effects of the subjective norm (β = − 0.062, *p* > 0.05) on vaccine hesitancy was not significant, inferring that knowledge about vaccine significantly fully mediates the relationship between subjective norm and vaccine hesitancy.

**Table 7:** The mediating effects of knowledge about vaccines on the relationship between TPB constructs and vaccine hesitancy.

Path	β	Stdβ	c	95% CI [LCI, UCI]	*t*	*p*
**(a) Mediation effect of knowledge about vaccine on the relationship between negative attitude towards vaccine and vaccine hesitancy**						
Total effect	0.124	0.374	0.042	0.299, 0.476	11.8689	0.012
Direct effect	0.120	0.362	0.042	0.280, 0.458	11.6342	0.014
Indirect effect	0.004	0.011	0.008	− 0.001, 0.032	–	0.048
**(b) Mediation effect of knowledge about vaccine on the relationship between subjective norms and vaccine hesitancy**						
Total effect	− 0.020	− 0.022	0.009	− 0.044, − 0.008	− 2.4611	0.004
Direct effect	0.000	0.000	0.000	0.000, 0.000	–	–
Indirect effect	− 0.020	− 0.022	0.009	− 0.044, − 0.008	–	0.004
**(c) Mediation effect of knowledge about vaccine on the relationship between perceived behavioural control and vaccine hesitancy**						
Total effect	− 0.264	− 0.138	0.043	− 0.216, − 0.038	− 3.1239	0.005
Direct effect	− 0.204	− 0.106	0.043	− 0.178, − 0.005	− 2.2515	0.006
Indirect effect	− 0.060	− 0.031	0.009	− 0.054, − 0.017	–	0.004
**(d) Mediation effect of knowledge about vaccine on the relationship between anticipated regret and vaccine hesitancy**						
Total effect	− 0.157	− 0.115	0.026	− 0.168, − 0.067	− 3.9033	0.008
Direct effect	− 0.127	− 0.092	0.026	− 0.146, − 0.043	− 3.1454	0.008
Indirect effect	− 0.031	− 0.022	0.007	− 0.037, − 0.009	–	0.016

All parameters obtained from the AMOS output with the TPB dimensions: Negative attitude towards vaccine, subjective norms, perceived behavioural control and anticipated regret; β—unstandardised beta co-efficient; Stdβ—standardised beta co-efficient; SE—standard error; 95% CI—95% confidence interval with lower bounds and upper bounds [LCI, UCI]; t—t value, *p*—shows the significant level.

**Figure 3:**
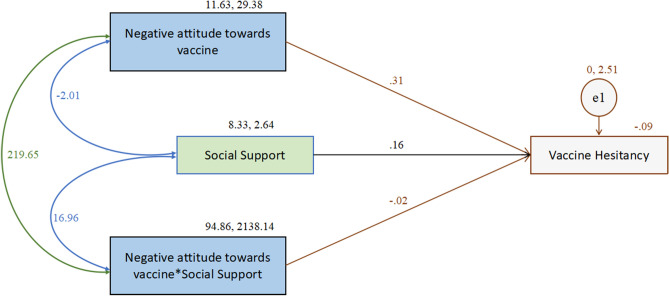
The moderating effect of social support on the relationship between negative attitude towards vaccine and vaccine hesitancy.

Moderation analyses were computed to investigate the effects of social support on the constructs. While social support showed no moderating effects between HBM constructs and hesitancy, the analysis indicated that social support significantly moderates the relationship of negative attitude towards vaccine on vaccine hesitancy (Table 8, Fig. 3). The effect of negative attitude towards vaccine (β = 0.313, Std β = 0.943, SE = 0.046, *t* = 6.863, *p* < 0.001), social support (β = 0.157, Std β = 0.142, SE = 0.075, *t* = 2.088, *p* < 0.05), and their interaction (β = − 0.020, Std β = − 0.524, SE = 0.005, *t* = − 3.813, *p* < 0.001) were all found to have significant effects on hesitancy.

**Table 8:** The moderating effect of social support on the relationship between negative attitude towards vaccine and vaccine hesitancy.

Path	β	Stdβ	SE	*t*	*p*
Effect of social support on vaccine hesitancy	0.157	0.142	0.075	2.088	**
Effect of negative attitude towards vaccine on vaccine hesitancy	0.313	0.943	0.046	6.863	***
Effect of “social support** × **negative attitude towards vaccine” on vaccine hesitancy	− 0.020	− 0.524	0.005	− 3.813	***

Social support—moderating variable; negative attitude towards vaccine—one of the TPB dimensions; β—unstandardised beta co-efficient; Stdβ—standardised beta co-efficient; SE—standard error; t—t value, *p*—shows the significant level; All parameters obtained from the AMOS output.

***p < 0.001; **p < 0.05.

**Figure 4:**
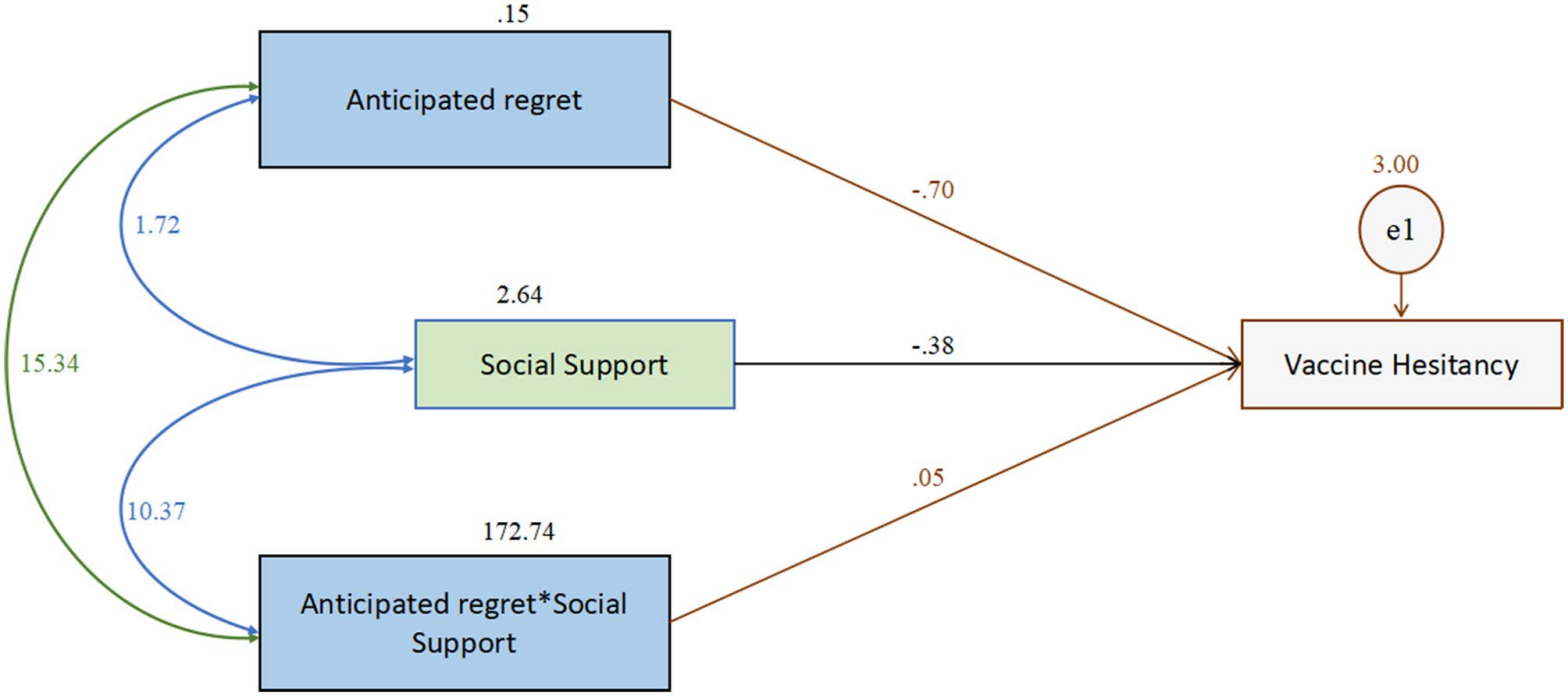
The moderating effect of social support on the relationship between anticipated regret and vaccine hesitancy.

Likewise, (Table 9, Fig. 4) the effect of anticipated regret (β = − 0.700, Std β = − 1.079, SE = 0.217, *t* = − 3.219, *p* < 0.001), social support (β = − 0.381, Std β = − 0.263, SE = 0.091, *t* = − 4.169, *p* < 0.001), and their interaction (β = 0.053, Std β = 0.067, SE = 0.025, *t* = 2.156, *p* < 0.05) were also found to have significant effect on vaccine hesitancy, thus indicating that social support significantly moderates between anticipated regret and vaccine hesitancy.

**Table 9:** The moderating effect of social support on the relationship between anticipated regret and vaccine hesitancy.

Path	β	Stdβ	SE	*t*	*p*
Effect of social support on vaccine hesitancy	− 0.381	− 0.263	0.091	− 4.169	***
Effect of anticipated regret on vaccine hesitancy	− 0.700	− 1.079	0.217	− 3.219	***
Effect of “social support** × **anticipated regret” on vaccine hesitancy	0.053	0.067	0.025	2.156	**

Social support—moderating variable; anticipated regret—one of the TPB dimensions; β—unstandardised beta co-efficient; Stdβ—standardised beta co-efficient; SE—standard error; t—t value, *p*—shows the significant level; All parameters obtained from the AMOS output.

***p < 0.001; **p < 0.05.

## Discussion

Although a few studies in India have reported the prevalence of vaccine hesitancy, there is still a shortage concerning the psychological factors that possibly influence vaccine hesitancy in India. The current study is one of a kind that tried to explore and investigate the factors predicting vaccine hesitancy using HBM and TPB models among a few Indian participants.

The findings from the present study indicated that, from the overall collected samples, vaccine hesitancy behaviour was not too high. A similar result was found in the Indian context by Jain et al.^32^ that emphasized the association of awareness of the COVID-19 vaccine with lower rates of vaccine-hesitant behaviours. Additionally, individuals are highly supportive of getting vaccinated, thus highlighting a positive outlook toward vaccine efficacy. This shows a low vaccine hesitancy from the participants of this study. As opposed to previous findings obtained in certain Indian surveys^24^, the current study plan was executed from July 2021, during which the vaccination drive was at a full-fledge; and as on 31st of July, 2021 India had crossed 46.15 crores (461.5 million) doses^32^. During this period, mass vaccination was made available for the citizens for free in Government hospitals across India which was possible to avail immediately for elderly and middle adults, but young adults had to wait to schedule their vaccine dose^33^. Nevertheless, the study does yield results that converge towards vaccine hesitant behaviours. Findings indicated that despite the free vaccination drive, social mobilization plan and campaign^25,26^, participants were still unwilling to get vaccinated. A similar study finding by Agarwal and Naha^34^ revealed that the first and second phases consisted of vulnerable and elderly individuals with co-morbidities, and their deaths were associated with vaccine side-effects. Vaccine hesitancy, therefore, was highest in India during the third and fourth phase of the vaccination drive due to the fear that was generated among the individuals concerning the safety of the vaccine. The study findings indicated that vaccine hesitancy still existed mainly among the rural and illiterate population despite the campaigns^34^.

While in the process to investigate the predictor power, the findings show that the integrative model of HBM and TPB was found to be good predictors of vaccine hesitancy. Further, the constructs from the HBM model highlight that an increase in the perceived susceptibility, perceived benefits and self-efficacy would reduce vaccine hesitancy. At the same time, an increase in perceived barriers would increase vaccine hesitancy. Similar contributions were highlighted from previous studies that emphasize the predictive power of perceived benefits and barriers, i.e., individuals tend to be less hesitant towards availing vaccine when they perceive benefits out of it, and they can avail it with more convenience and no barriers^8,14,16^. Badr et al.^35^ also revealed that individuals who perceived that they could have a greater risk of acquiring COVID-19 and those who perceived greater benefits with vaccination showed lower vaccine hesitancy. However, in the current study, perceived severity showed no significant predictions on vaccine hesitancy. Rastogi et al.^36^ reported individual’s low perceived severity towards COVID-19  as one of the main reasons for non-compliance or poor compliance of protective measures or behaviours in India. Lin et al.^37^ also had a similar observation along with Lau et al.^38^, and Coe et al.^39^ Although the current study indicates that the severity of the COVID-19 virus would not affect an individual’s decision to get immunized, their belief of contracting the COVID-19 virus would definitely influence the individuals’ vaccination intake. Moreover, the benefits they perceive as an outcome and their belief in getting vaccinated would influence vaccine behaviour. Likewise, the findings of the analysis of the constructs from the TPB model indicated that an increase in anticipated regret would reduce the vaccine hesitancy. Also, in the current study, a higher value of attitude towards vaccine denotes a negative attitude towards a vaccine. Thus, an increase in a negative attitude towards vaccines indicates an increase in vaccine hesitancy. These findings are similar to previous research findings^12,16,17^, where in order to avail vaccination, one must have a positive attitude towards it. Creating awareness by inculcating regret could also play a significant role in promoting vaccine behaviours. Hossain et al.^16^ indicated that anticipated regret evidently contributed to reducing vaccine hesitancy and, therefore, should be incorporated in awareness programs that would indicate regret if one doesn’t get immunized^16^.

Furthermore, the present study attempted to investigate the mediating and moderating effects of knowledge about vaccines and social support using AMOS, which has not been explicitly explored in India. Mediation analysis of knowledge about vaccine between the HBM constructs and vaccine hesitancy indicated that perceived benefits, perceived barriers, cues to action, and self-efficacy directly affected vaccine hesitancy. However, in this mediation analysis, it was found that perceived susceptibility and severity had no impact. Similar findings by Jones et al.^40^ and Chen^41^ observed perceived susceptibility and perceived severity being the least powerful predictor across most studies.

Interestingly, self-efficacy showed the highest influence on hesitancy via knowledge about vaccine, indicating that the individual’s belief in their ability to get vaccinated is highly influenced by their understanding of vaccine, which reduces vaccine hesitancy. Consequently, the findings generated in this study could be incorporated or compared with the three different categories viz., *complacency, convenience* and *confidence* of the 3C model. It was determined that hesitancy was best explained through these three categories^7^. The current study found that hesitancy would be reduced if individuals inculcated a positive attitude towards vaccine or perceived more positive aspects or benefits while getting vaccinated. This ensures *complacency*, which emphasizes that individuals would become hesitant if they perceive the risk involved with vaccination. Likewise, *convenience* suggests that the availability of vaccines, location, other physical constructs could play a vital role in an individual’s decision-making process. Our study implicated that perceived barriers exert a positive variance on vaccine hesitancy, thus indicating the importance of such physical constructs in influencing an individual’s decision to get vaccinated. An individual’s *confidence* in the perceived benefits would also aid in reducing the hesitancy behaviour. These findings further acknowledge the 3C models’ categories and it’s relevance to date^7,42^.

In addition to the above findings, the mediation analysis also emphasizes that, because the individuals are aware of the vaccine or have adequate knowledge about the vaccine, they favour vaccination and thus also validate the other finding i.e., low rates of vaccine hesitancy. Moreover, the analysis of TPB constructs also indicates that having adequate knowledge about vaccines aids in vaccine hesitancy, i.e., knowledge about vaccines explains the relationship between the TPB constructs and vaccine hesitancy. An interesting finding i.e., the full mediation of subjective norm via knowledge was noted, implicating that mediation analysis showed no direct influence of subjective norm. However, it had strong indirect effects on vaccine hesitancy , thus indicating that the individuals conform to their social and family groups because of the knowledge about the vaccine that they presume the social groups possess. These findings could be supported by Eitze et al.^43^ and Ashkenazi et al.^44^ studies that emphasized the role of knowledge in reducing vaccine hesitancy or vaccine intention behaviour.

Moreover, findings also revealed the role of social support in reducing vaccine hesitancy. While none of the HBM constructs showed significant interacting effects with social support, of the TPB constructs, it was found that negative attitude toward a vaccine and anticipated regret had interacting effects with social support. Although these have direct influences on hesitancy, the presence of social support cannot be ignored. Likewise, in the presence of social support, the effect of negative attitude toward a vaccine and social support leads to increased vaccine hesitancy, but their interaction reduces vaccine hesitancy. Contrarily, the impact of anticipated regret and social support leads to a decrease in vaccine hesitancy. However, their interaction leads to an increase in vaccine hesitancy. This revealed that any form of social support could interplay with the regret individuals may anticipate, thus nullifying it and increasing hesitancy.

Throughout the study, the exploration of vaccine hesitancy has been investigated well with the help of HBM, TPB, knowledge about vaccines, and social support. The findings could be incorporated in creating awareness and persuasive information for the general public.

## Conclusion

The findings of this study revealed the prevalence rate of vaccine hesitancy in the Indian population. Individuals are willing to get vaccinated and even encourage their family and friends to get vaccinated. Knowledge and awareness regarding the vaccine has been found to play a vital role in low rates of vaccine hesitancy. The study emphasizes the role of social media platforms and other sources of information that act as cues to reduce vaccine hesitancy  indirectly. Additionally, instead of creating fear regarding the severity, if authorities and health workers highlight the positive outcomes and benefits of getting vaccinated and their susceptibility towards the COVID-19 virus, the hesitancy behaviour will be significantly reduced. Moreover, inducing self-efficacy and removing barriers could also mitigate the vaccine-hesitant behaviours.

Furthermore, creating environments where individuals would conform to the social pressure rather than directly coercing would influence individuals to get vaccinated. Social support has been found to have a moderating role, provided that the support promotes vaccination behaviour. Likewise, communicating persuasive messages that highlight the adverse effects of regret could influence individuals to get immunized. Overall, this study suggests that the HBM and the TPB model could explain the psychological influences of vaccine hesitancy among the Indian respondents. The findings highlight how knowledge about a vaccine could affect the hesitation and how social support could play a role in getting vaccinated.

## Strengths and limitations

This study is among the few that incorporated HBM, TPB, knowledge about vaccines, and social support to examine the effects and variations exerted on vaccine hesitancy in India. The findings could be utilized to design interventions that promote vaccination behaviours and reduce hesitancy. One of the main limitations of this study is the sample characteristics. The researchers couldn’t use the randomized technique to collect the data, and therefore the findings may not represent the entire nation.

## References

[1] World Health Organization. *WHO Coronavirus (COVID-19) Dashboard*. 2021. Retrieved on 13th August 2021 from https://covid19.who.int/?gclid=CjwKCAjwjdOIBhA_EiwAHz8xm9rz2Bcv52vSJy1m_ARSTgEOCvYjl-WWkMUfafUsA_Pg_W37yOF5uBoCQhYQAvD_BwE

[2] Sharun K, Dhama K (2021). India’s role in COVID-19 vaccine diplomacy. J. Travel Med..

[3] Bagcchi S (2021). The World's largest COVID-19 vaccination campaign. Lancet Infect. Dis..

[4] Lancet T (2021). India's COVID-19 emergency. Lancet.

[5] Surapaneni KM, Kaur M, Kaur R, Grover A, Joshi A (2021). The impact of COVID-19 vaccine communication, acceptance, and practices (CO-VIN-CAP) on vaccine hesitancy in an Indian setting: Protocol for a cross-sectional study. JMIR Res. Protoc..

[6] MacDonald NE (2015). Vaccine hesitancy: Definition, scope and determinants. Vaccine.

[7] WHO EURO Working Group on Vaccine Communications. Istanbul, Turkey 2011; October 13–14.

[8] Shmueli L (2021). Predicting intention to receive COVID-19 vaccine among the general population using the health belief model and the theory of planned behavior model. BMC Public Health.

[9] Rosenstock IM (1974). The health belief model and preventive health behavior. Health Educ. Monogr..

[10] Green EC, Murphy EM, Gryboski K, Sweeny K, Robbins ML, Cohen LM (2020). The health belief model. The Wiley Encyclopedia of Health Psychology.

[11] Keith T (2011). Conceptual analysis of behavioral theories/models: Application to financial behavior. Eur. J. Soc. Sci..

[12] Guidry JP (2021). Willingness to get the COVID-19 vaccine with and without emergency use authorization. Am. J. Infect. Control.

[13] Champion VL, Skinner CS, Glanz K, Rimer BK, Viswanath K (2008). The health belief model. Health Behavior and Health Education: Theory, Research, and Practice.

[14] Lin CY (2020). Using an integrated social cognition model to predict COVID-19 preventive behaviours. Br. J. Health Psychol..

[15] Sandberg T, Conner M (2008). Anticipated regret as an additional predictor in the theory of planned behaviour: A meta-analysis. Br. J. Soc. Psychol..

[16] Hossain MB (2021). Health belief model, theory of planned behavior, or psychological antecedents: What predicts COVID-19 vaccine hesitancy better among the Bangladeshi adults?. Front. Public Health.

[17] Myers LB, Goodwin R (2012). Using a theoretical framework to determine adults' intention to vaccinate against pandemic swine flu in priority groups in the UK. Public Health.

[18] Behavioral Change Models. Retrieved on 28th February 2022 from https://sphweb.bumc.bu.edu/otlt/mph-modules/sb/behavioralchangetheories/BehavioralChangeTheories_print.html

[19] Taylor, D. *et al*. *A Review of the use of the Health Belief Model (HBM), the Theory of Reasoned Action (TRA), the Theory of Planned Behaviour (TPB) and the Trans-Theoretical Model (TTM) to study and predict health related behaviour change. National Institute for Health and Clinical Excellence* (2007); Retrieved from https://warwick.ac.uk/fac/sci/med/study/ugr/mbchb/phase1_08/semester2/healthpsychology/nice-doh_draft_review_of_health_behaviour_theories.pdf

[20] Wong CY, Tang CSK (2005). Practice of habitual and volitional health behaviors to prevent severe acute respiratory syndrome among Chinese adolescents in Hong Kong. J. Adolesc. Health.

[21] Cheng C, Ng AK (2006). Psychosocial factors predicting SARS-preventive behaviors in four major SARS-affected regions. J. Appl. Soc. Psychol..

[22] Alsulaiman SA, Rentner TL (2018). The health belief model and preventive measures: A study of the ministry of health campaign on coronavirus in Saudi Arabia. J. Int. Crisis Risk Commun. Res..

[23] Arora, H. India has a vaccine hesitancy challenge. *The Indian Express* (2021). Retrieved on 31st July, 2021 from https://indianexpress.com/article/opinion/india-has-a-vaccine-hesitancy-challenge-7388907/

[24] The Wire. Vaccine hesitancy puts India's gains against coronavirus at risk. *The Live Mint News* (2021). Retrieved on 31st July 2021 from https://www.livemint.com/news/india/vaccine-hesitancy-puts-india-s-gains-against-coronavirus-at-risk-11624252466615.html

[25] Ahuja, A. & Bhaskar, S. From creating role models to vaccinating at home, here’s how J&K’s Bandipora district has rolled out COVID vaccination. *NDTV* (2021). Retrieved on February 28 from https://swachhindia.ndtv.com/from-creating-role-models-to-vaccinating-at-home-heres-how-jks-bandipora-district-has-rolled-out-covid-vaccination-60450/

[26] Singh, P., Dhawan, V. & Rishi, G. *COVID-19 Communication Strategy 2020*. Ministry of Health and Family Welfare, Government of India. Retrieved on 28th February, 2022 from https://www.mohfw.gov.in/pdf/Covid19CommunicationStrategy2020.pdf

[27] Kim S, Kim S (2020). Analysis of the impact of health beliefs and resource factors on preventive behaviors against the COVID-19 Pandemic. Int. J. Environ. Res. Public Health.

[28] Kotta I, Kalcza-Janosi K, Szabo K, Marschalko EE (2021). Development and validation of the multidimensional COVID-19 vaccine hesitancy scale. Hum. Vaccines Immunother..

[29] Field A (2013). Discovering Statistics Using IBM SPSS Statistics.

[30] Nimon KF (2012). Statistical assumptions of substantive analyses across the general linear model: A mini-review. Front. Psychol..

[31] Streiner DL (2005). Finding our way: An introduction to path analysis. Can. J. Psychiatry..

[32] Jain J (2021). COVID-19 vaccine hesitancy among medical students in India. Epidemiol. Infect..

[33] PIB’S 32Bulletin On COVID-19. *Press Information Bureau*. Ministry of Information and Broadcasting. Government of India. Retrieved on 31st July, 2021 from https://pib.gov.in/PressReleaseIframePage.aspx?PRID=1741093

[34] Agarwal SK, Naha M (2021). COVID-19 vaccine hesitancy in india: An exploratory analysis. MedRxiv.

[35] Badr H, Zhang X, Oluyomi A, Woodard LD, Adepoju OE, Raza SA, Amos CI (2021). Overcoming COVID-19 vaccine hesitancy: Insights from an online population-based survey in the United States. Vaccines.

[36] Rastogi T, Awasthi S, Khare R, Prasad M, Sami G, Verma VK (2022). Perceptions and practices of COVID-19 protective measures among the general public of North India. Clin. Epidemiol. Glob. Health.

[37] Lin Y, Hu Z, Zhao Q, Alias H, Danaee M, Wong LP (2020). Understanding COVID-19 vaccine demand and hesitancy: A nationwide online survey in China. PLoS Negl. Trop. Dis..

[38] Lau JT, Yeung NC, Choi KC, Cheng MY, Tsui HY, Griffiths S (2010). Factors in association with acceptability of A/H1N1 vaccination during the influenza A/H1N1 pandemic phase in the Hong Kong general population. Vaccine.

[39] Coe AB, Gatewood SB, Moczygemba LR (2012). The use of the health belief model to assess predictors of intent to receive the novel (2009) H1N1 influenza vaccine. Innov. Pharm...

[40] Jones CL, Jensen JD, Scherr CL, Brown NR, Christy K, Weaver J (2015). The health belief model as an explanatory framework in communication research: Exploring parallel, serial, and moderated mediation. Health Commun..

[41] Chen H (2021). Health belief model perspective on the control of COVID-19 vaccine hesitancy and the promotion of vaccination in China: Web-based cross-sectional study. J. Med. Internet Res..

[42] MacDonald, N. E. *Report of the Sage working group on vaccine hesitancy.* 2014. Retrieved from https://www.who.int/immunization/sage/meetings/2014/october/1_Report_WORKING_GROUP_vaccine_hesitancy_final.pdf

[43] Eitze S, Heinemeier D, Schmid-Küpke NK, Betsch C (2021). Decreasing vaccine hesitancy with extended health knowledge: Evidence from a longitudinal randomized controlled trial. Health Psychol..

[44] Ashkenazi S, Livni G, Klein A, Kremer N, Havlin A, Berkowitz O (2020). The relationship between parental source of information and knowledge about measles/measles vaccine and vaccine hesitancy. Vaccine.

